# Management of elderly ulcerative colitis in Japan

**DOI:** 10.1007/s00535-019-01580-y

**Published:** 2019-04-20

**Authors:** Masaaki Higashiyama, Akira Sugita, Kazutaka Koganei, Kenji Wanatabe, Yoko Yokoyama, Motoi Uchino, Masakazu Nagahori, Makoto Naganuma, Shigeki Bamba, Shingo Kato, Ken Takeuchi, Teppei Omori, Tomohisa Takagi, Satohiro Matsumoto, Mitsuo Nagasaka, Shintaro Sagami, Kazuya Kitamura, Takehiko Katsurada, Ken Sugimoto, Noritaka Takatsu, Masayuki Saruta, Toshiyuki Sakurai, Kazuhiro Watanabe, Shiro Nakamura, Yasuo Suzuki, Ryota Hokari

**Affiliations:** 1 Department of Internal Medicine, National Defense Medical College, 3-2 Namiki, Tokorozawa, Saitama 359-8513 Japan; 2 Inflammatory Bowel Disease Center, Yokohama Municipal Citizen’s Hospital, Yokohama, Kanagawa Japan; 3 Department of Intestinal Inflammation Research, Hyogo College of Medicine, Nishinomiya, Hyogo Japan; 4 Department of Inflammatory Bowel Disease, Division of Surgery, Hyogo College of Medicine, Nishinomiya, Hyogo Japan; 5 Department of Gastroenterology and Hepatology, Tokyo Medical and Dental University, Tokyo, Japan; 6 Division of Gastroenterology and Hepatology, Department of Internal Medicine, Keio University School of Medicine, Tokyo, Japan; 7 Division of Clinical Nutrition, Shiga University of Medical Science, Otsu, Shiga Japan; 8 Department of Gastroenterology and Hepatology, Saitama Medical Center, Saitama Medical University, Saitama, Japan; 9 Division of Gastroenterology and Hepatology, Department of Internal Medicine, Toho University Sakura Medical Centre, Sakura, Chiba Japan; 10 Institute of Gastroenterology, Tokyo Women’s Medical University, Tokyo, Japan; 11 Molecular Gastroenterology and Hepatology, Graduate School of Medical Science, Kyoto Prefectural University of Medicine, Kyoto, Japan; 12 Department of Gastroenterology, Saitama Medical Center, Jichi Medical University, Saitama, Japan; 13 Department of Gastroenterology, Fujita Health University School of Medicine, Toyoake, Aichi Japan; 14 Center for Advanced IBD Research and Treatment, Kitasato University Kitasato Institute Hospital, Tokyo, Japan; 15 Department of Gastroenterology, Kanazawa University Hospital, Kanazawa, Ishikawa Japan; 16 Department of Gastroenterology and Hepatology, Hokkaido University Graduate School of Medicine, Sapporo, Hokkaido Japan; 17 First Department of Medicine, Hamamatsu University School of Medicine, Hamamatsu, Shizuoka Japan; 18 Department of Gastroenterology, Fukuoka University Chikushi Hospital, Chikushino, Fukuoka Japan; 19 Division of Gastroenterology and Hepatology, Department of Internal Medicine, The Jikei University School of Medicine, Tokyo, Japan; 20 Department of Surgery, Tohoku University Graduate School of Medicine, Sendai, Miyagi Japan; 21 Inflammatory Bowel Disease Center, Toho University Sakura Medical Centre, Sakura, Chiba Japan

**Keywords:** Ulcerative colitis, Inflammatory bowel disease, Elderly, Management

## Abstract

Japan has the largest aging society, where many elderly people have intractable diseases including ulcerative colitis (UC). Along with the increasing total number of UC patients, the number of elderly UC patients has also been increasing and will continue to do so in the future. Although the clinical features and natural history of UC in the elderly have many similarities with UC in the non-elderly population, age-specific concerns including comorbidities, immunological dysfunction, and polypharmacy make the diagnosis and management of elderly UC challenging compared to UC in non-elderly patients. Based on increasing data related to elderly UC patients from Japan, as well as other countries, we reviewed the epidemiology, clinical course, differential diagnosis, management of comorbidities, surveillance, medical therapy, and surgery of UC in the elderly.

## Introduction

Japan has the largest aging society with an average life span of more than 80 years in 2017 (81.09 years in males and 87.26 years in females) [1], and those who are 65 years or older comprise 27.7% of the society [2]. In this graying society, the elderly population faces many problems including comorbidities, immunological dysfunction, and polypharmacy.

There are many elderly people with intractable diseases including inflammatory bowel disease (IBD), comprising Crohn’s disease (CD) and ulcerative colitis (UC), a chronic immunologically mediated disease at the intersection of complex interactions between genetics, environment, and gut microbiota [3]. While early life events such as birth, breastfeeding, and exposure to antibiotic and later childhood events are considered potential risk factors [4], IBD can present at any age. In Japan, the number of UC patients has been increasing year by year. Estimates based on the issued numbers of certificates of recipients of medical service and certificates of registration in 2013 showed that there are over 160,000 patients with UC (approximately 100 cases per 100,000) [5]. Approximately 10% of newly registered UC patients were 65 years old or more according to a nationwide study [6], suggesting that the number of elderly UC patients has also been increasing and will continue to do so in the future.

Although the clinical features and natural history of elderly UC have many similarities with non-elderly UC, the diagnosis and management of elderly UC patients are challenging compared to non-elderly UC patients owing to age-specific concerns. Considering that elderly UC patients have higher rates of cardiac diseases, infections, malignancies, and UC-related mortalities, elderly UC patients need to be treated more carefully than non-elderly UC patients [7, 8]. There are already some reviews or guidelines for IBD patients from Asia including Japan [9, 10], but no manuscript specific for the elderly. Based on increasing data related to elderly UC patients from Japan, as well as other countries, in the present study, we reviewed the epidemiology, clinical course, differential diagnosis, management of comorbidities, surveillance, medical therapy, and surgery of elderly UC.

## Diagnosis and epidemiology in elderly UC patients

### Definition of elderly UC patients

The age at which being elderly is defined as is not universally accepted. Elderly people are more likely to have infections, cardiovascular diseases, impaired glucose tolerance, and malignancies compared to young people. The comorbidities and frailties should be considered in defining the age of elderly UC patients, which is often defined as “over 60 years” [7, 8, 11–17] or “over 65 years” [6, 18–22] for convenience, but there is no universal definition currently. Most developed countries have set the chronological age of over 65 years as the definition of an elderly person. Similarly, in Japan, elderly people are often defined as having a chronological age of 65 years or over. However, considering the long life expectancy of the Japanese and its socio-economic influence, some people say that the definition of the elderly should be an individual who is older than 75 years [23]. In this manuscript, we have described the definition of elderly age or older/oldest group age, or article type (review, meta-analysis, guideline, and so on) after each reference in the Reference list.

The clinical course in elderly UC patients is reported to be different between elderly patients with late age onset of UC (elderly-onset UC, EOUC) and long-standing elderly UC (LEUC), that is, those who were first diagnosed as having UC at a younger age [22]. Therefore, it is necessary to take the age of onset and the disease duration into account when making decisions regarding the treatment of elderly UC patients.

### Epidemiology of elderly-onset UC patients

Shi et al. reported that the number of EOUC patients in Hong Kong increased from 0.1/100,000 in 1991 to 1.3/100,000 in 2010 [17]. In Japan, the number of UC patients has been increasing, with patients more likely to develop the disease between their late 10s and early 30s [9]. Although there are no published statistical data, the age of patients gradually shifts to an elderly population in the aging society and opportunities to encounter elderly UC patients seem to be increasing [9].

The onset of UC is most frequent between 30 and 40 years of age, but previous studies have shown a second peak of incidence in the elderly population [24, 25]. Stowe et al. showed that the incidence in the elderly population (between the ages of 60 and 80 years) was higher than that in the population aged 40–50 years. However, this trend has not been observed in other cohort studies [26, 27]. According to a study by Steven et al., the percentage of newly diagnosed elderly UC patients in the Netherlands was 18.3% in 1991, which increased to 25.9% in 2010 [12].

In a systematic review from an Asian country, Prideaux et al. showed that the age at onset of UC was higher than that of CD, with the average age at onset of UC being 35–44 years [28]. Asakura et al. investigated the number of UC patients in Japan in 5-year age intervals [29] and found that the peak age of incidence was 30–35 years followed by no bimodal peak in the elderly. Takahashi et al. compared the age distribution of UC patients who were diagnosed before 2000 and after 2001. They found that the ratio of EOUC patients increased from 6.3% in those diagnosed before 2000 to 25.9% in those diagnosed after 2001. They also determined that the average age at UC onset was increasing and that a second peak in bimodal distribution of onset shifted to older age [30]. Song et al. reported that the percentage of elderly patients among newly diagnosed patients had increased from 3.9% in 1977–1999 to 9.7% in 2008–2014 [31]. Although the cause the increasing incidence of EOUC in several regions and ethnicities remains unclear, Takahashi et al. reported that past smoking habit is a possible risk factor (OR 2.93) for elderly-onset UC [30]. Araki et al. reported that more than half of the IBD patients (51.1%) experienced seasonal exacerbation of IBD, and winter was the most common season for disease exacerbation. However, seasonality of disease onset and exacerbation was observed in young-onset patients (≤ 40 years old), but not in elderly-onset patients [32].

### Differential diagnosis in elderly patients with UC

As shown in IBD guidelines in Japan [9], it is often necessary to distinguish UC from infectious enterocolitis, especially *Campylobacter*, enteroinvasive *Escherichia coli*, and amoebic dysentery. In addition, immunological dysfunction and lifestyle-related diseases should be taken into consideration to differentiate diagnoses along with the aging. Additionally, since the aging increases the prevalence of lifestyle-related diseases including arteriosclerosis, high blood pressure, diabetes, ischemic heart diseases, and stroke, lifestyle-related diseases and treatment induce colitis necessary to be differentiated from UC (Table 1).

**Table 1:** Differential diagnosis for ulcerative colitis in the elderly patients

**Infection-related diseases**
*Clostridium difficile* (*Clostridioides difficile*)-associated diarrhea (CDAD)
Intestinal tuberculosis
Cytomegalovirus colitis
**Cancer-related diseases**
Radiation colitis
UC-like colitis induced by immune checkpoint inhibitors
**Lifestyle-related diseases**
NSAID-induced intestinal disorder
Microscopic colitis
Ischemic colitis
Segmental colitis associated with diverticulosis (SCAD)
Solitary rectal ulcer syndrome (SRUS)

*Clostridium difficile* (renaming as *Clostridioides difficile* in 2016)-associated diarrhea (CDAD) sometimes occurs in patients using antibiotics or leukopenic patients induced by anti-cancer drugs. The important clinical risk factors for CDAD are advanced age (over 70 years) and long hospital stay (more than 20 days) [33]. Since pseudomembranes, the typical indication of CDAD, are not detected in any of the patients using immunosuppressive agents, bacterial examination is essential when UC becomes exacerbated even in the absence of pseudomembranes [34], in which case ELISA examination of *Clostridium difficile* toxin is one of the useful diagnostic tools. Cytomegalovirus (CMV) colitis often occurs in immunodeficiency patients and in elderly patients [35, 36]. Radiation colitis shows UC-like endoscopic appearances in the cancer patients treated with radiation therapy. Colonic mucosa is a radiation-sensitive organ and radiation-induced injuries often occur in the rectum [37]. Recently, cancer patients have been prescribed immune checkpoint inhibitors, with 1 of the complications being UC-like colitis [38, 39]. The increasing chronic usage of non-steroidal anti-inflammatory drugs (NSAIDs) for orthopedic diseases and proton pump inhibitors (PPIs) for the treatment of gastroesophageal reflux and the prevention of gastrointestinal bleeding due to anti-coagulative drugs sometimes trigger NSAID-induced enteropathy and microscopic colitis, respectively [40, 41]. Patients with microscopic colitis present with chronic diarrhea accompanied by redness, longitudinal ulcers, and scars in the colon. Ischemic colitis often occurs in the elderly patients because of the arteriosclerosis [42]. Idiopathic colitis in the left side colonic diverticulosis is called “segmental colitis associated with diverticulosis, SCAD” [43, 44]. SCAD often occurs in elderly patients and is difficult to differentiate it from UC owing to the similarities including endoscopic findings, histology, and good response to mesalazine [45]. Solitary rectal ulcer syndrome (SRUS) is considered to be induced by the mucosal prolapse of the anterior wall by straining during defecation [46]. SRUS results in ulcers and elevated lesions in the rectum, and its pathological features show fibromuscular obliteration. Some cases of SRUS complicated by UC have also been reported [47].

## Natural history, disease course, and disease type in elderly UC patients

### Symptoms, severity, disease type/extent of elderly UC patients

Major clinical symptoms of UC include hematochezia, diarrhea, abdominal pain, weight loss, and fever and the incidence and severity of these symptoms in EOUC and non-elderly-onset UC (NEOUC) are different according to several reports. In Japan, severity was significantly higher in EOUC patients than in NEOUC patients [6]. However, the disease activity was higher in NEOUC patients than in EOUC patients in France, and Turkey [14, 15]. Meanwhile, there was no difference in disease severity between EOUC and NEOUC in Hong Kong [17]. Taken together, there are no remarkable changes in symptomatic severities between EOUC and NEOUC [7].

In terms of disease distribution, there is a significantly, but only a slightly, wider distribution in Japan of EOUC (EOUC; proctitis:left-sided colitis:pancolitis = 19.5%:34.2%:46.3%, NEOUC; proctitis:left-sided colitis:pancolitis = 23.9%:31.7%:44.5%) [6]. Meta-analysis showed a higher rate of left-sided colitis in EOUC but the rates of disease distributions were almost the same in proctitis and pancolitis between EOUC and NEOCU [8].

In summary, no significant difference was observed in disease severity and distribution between EOUC and NEOUC according to several reports from different countries.

### Differences between long-standing elderly UC and elderly-onset UC

Distinguishing between LEUC patients with a longer disease duration and EOUC patients with a shorter disease duration is important as those who have been affected for longer have a lesser frequency of surgery and admission.

The surgical rate in UC patients is higher during the first 2 years after disease onset, which decreases gradually thereafter [48]. Therefore, if we compare the surgical rate and hospital admission rate of elderly EOUC and LEUC patients of the same age, it would be higher in EOUC patients because disease duration is shorter in EOUC than in LEUC. In a study by Matsumoto et al., the rate of steroid use was recorded in 58% of EOUC patients, but 0% of the LEUC patients during disease exacerbations. Resistance to or dependence on prednisolone was observed in 17% of EOUC patients. The rate of hospital admission due to exacerbation of UC was 25% and the rate of emergency surgery was 17% in EOUC patients. On the other hand, none of the LEUC patients were admitted to the hospital or underwent surgery during the study period [22].

It is well known that LEUC patients are at an increased risk for colorectal cancer because of the long duration of the disease [49, 50]. However, whether the risk for colorectal cancer increases more in older patients compared with younger patients with the same disease duration has not been examined in detail [51, 52]. On the other hand, surveillance for the development of colorectal cancer in patients with elderly-onset IBD is recommended based on the evidence that elderly-onset IBD is associated with an increased risk for colorectal cancer. Furthermore, the time to the development of colorectal cancer decreases by 0.154 point per 1 year increment of age [53]. The following statement was made by the European Crohn’s and Colitis Organization (ECCO) in 2017: “The screening program in the elderly should be balanced with disease severity, comorbidities, and life expectancy” [7].

### Hospitalization and surgery in elderly UC patients

Previously, studies have reported that the surgery rate of elderly UC patients is similar to that of non-elderly patients [12, 13, 18] with other studies reporting the converse, that the surgery rate of elderly patients is lower than that of non-elderly patients [14, 48]. The topical review of ECCO also says that the surgery rate of elderly UC patients does not differ from that of non-elderly patients. [7]. On the contrary, a recent meta-analysis targeting only EOUC reported a 1.36-fold higher surgical rate [8]. One of the possible reasons for the controversial results was that previous studies did not consider the onset of the disease or disease duration, which includes not only EOUC but LEUC, which is generally considered to be a less severe course of the disease [15]. Recently, similar results were reported from Asian countries [22, 54]. It has been reported that the surgery rate was significantly higher in EOUC patients than in NEOUC patients in a large-scale cohort study in Japan. One of the reasons why EOUC has a higher surgery rate is that EOUC patients have less chances of receiving strong immunosuppressive therapies owing to comorbidities and adverse events, such as infections [19, 55]. In addition, some reports showed that the perioperative mortality rate rises in emergency surgeries [16, 20], and life prognosis will be improved by early operations in elderly UC patients [56].

There are many reports that the hospitalization rate of elderly UC patients is significantly higher than that of non-elderly UC patients [7, 12, 17, 21, 22]. Moreover, it has been reported that the hospitalization rate is significantly higher in EOUC in a large-scale cohort study in Japan [6], probably due to more severe disease at onset, more comorbidities, and a higher risk for infectious events due to immunosuppressive therapies in elderly patients.

## Management for complications and comorbidities in elderly UC patients

### The significance of vaccination in elderly UC patients

In general, elderly people have an increased risk for infection based on a weakened immune system compared with non-elderly people [9]. Furthermore, UC patients treated with immunosuppressive agents such as corticosteroids, immunomodulators, or molecular-targeted agents are at an increased risk for infectious diseases [7, 57, 58], especially in UC patients treated with corticosteroids or multi-immunosuppressive agents [7, 55].

All UC patients should be tested for hepatitis B virus (HBV) at UC diagnosis to determine HBV status. Furthermore, an HBV vaccination is recommended in all HBV anti-HBc Ab-seronegative UC patients [59]. Many IBD patients were unaware of their past vaccinations or infections with the viruses causing varicella zoster, rubella, measles, or mumps [60], which are live vaccines (the recombinant subunit vaccine for herpes zoster virus (HZV) was approved in Japan at March 2018). Therefore, measuring the current levels of antibodies specific for these viruses is useful before administration of immunomodulators/biologics. Please refer to the previous guides for details about the vaccines mentioned above [59, 61].

Elderly UC patients (especially in Asia) treated with tofacitinib are at increased risk for herpes zoster [62]. The recombinant subunit vaccine for HZV (HZ/su, Shingrix^®^) [63], which is not a live vaccine and reduces the risk for herpes zoster among adults 70 years or older, was approved in Japan in March 2018. The effectiveness of the vaccine remains to be clarified in elderly UC patients. The pneumococcal vaccine and influenza vaccine, both inactivated vaccines, are often administered in elderly UC patients. The appropriate timing of pneumococcal vaccination should be considered when needed clinically except the regularly offered vaccination. On the other hand, annual influenza vaccinations are recommended in elderly UC patients [55, 61]. The inactivated vaccination can be administered even to UC patients who are treated with immunosuppressive agents such as anti-TNF; however, there is a possibility to reduce the immune response for influenza vaccinations in the elderly. [64]. The twice booster influenza vaccination is not effective with regard to increasing the immune response in adult IBD patients, unlike pediatric IBD patients [65].

### Influence of comorbidities

The proportion of IBD patients older than 65 years who have 3 or more comorbidities is 9.6%, which is higher than that of patients younger than 65 years (2.6%) [21]. In elderly IBD patients, the most common complications are diabetes (16.8%), heart diseases (such as myocardial infarction and congestive heart failure) (3.4%), chronic lung disease (21.4%), and cerebrovascular disease (3.1%). The risk for nephrotoxicity, particularly in elderly patients with IBD, which is complicated by heart failure or renal dysfunction, increases due to delayed excretion of 5-aminosalicylic acid (5-ASA). Therefore, it is unsustainable to administer a sufficient dose of 5-ASA and that could possibly lead to a deteriorated clinical course of IBD [11]. In addition, the risk for malignant tumors unrelated to IBD is higher in elderly IBD patients [21]. It has been reported that the proportion of cholangiocarcinoma is higher in solid cancers arising outside the gastrointestinal tract, and its risk increases with advancing age [66].

It is reported that the in-hospital mortality rate of elderly IBD patients (aged 65 and older) is higher than that of young IBD patients (19–64 years old), and the adjustment odds ratio of death rate also increases with the Charlson Comorbidity Index [21]. In addition, the presence of complications has a significant influence on the postoperative outcome in elderly IBD patients. Kaplan et al. [67] reported that among older surgical patients who underwent IBD-related surgery, the postoperative mortality rate increased as the number of complications increased. Complications of congestive heart failure, liver disease, thromboembolism, and renal disease are associated with a significant increase in mortality. Upon emergency surgery, the postoperative mortality rate of elderly people (65–80 years old) with 2 or more complications was the highest (20.6%). On the other hand, the mortality rate of patients with 2 comorbidities or fewer was 11.0%. In elective IBD surgery, the mortality rates of elderly patients with 2 or more comorbidities and less than 2 comorbidities were low, at 7.7% and 2.8%, respectively.

### Infectious diseases

Ananthakrishnan et al. analyzed an inpatient database in the US and presented that age was identified as an independent risk factor for hospitalization due to infectious complications such as pneumonia, sepsis, urinary tract infection, and *C. difficile* infection in IBD patients [68]. Toruner et al. later reported that increased age and immunosuppressive medications such as corticosteroids, especially when used in combination, were associated with an increased risk for opportunistic infections among IBD patients, and that those first evaluated older than the age of 50 years had significantly greater odds to have been diagnosed with an opportunistic infection (odds ratio 3.0; 95% CI 1.2–7.2) [55]. Furthermore, Naganuma et al. investigated risk factors of opportunistic infections in a 1-year cohort study and reported that an age of 50 years and older as well as use of immunomodulators were identified as independent risk factors of having opportunistic infections [57]. Furthermore, age was found to be independently associated with increased mortality. Cottone et al. reported that IBD patients older than 65 years treated with infliximab or adalimumab have a high rate of severe infections and mortality compared with younger patients or patients of the same age that did not receive these therapeutics [19]. Brassard et al. identified and investigated more than 3500 IBD patients who had developed IBD at age 66 and older from the Quebec healthcare database and reported that corticosteroid use was associated with serious infections [69].

Regarding the risk for *C. difficile* infection, Das et al. investigated if corticosteroids increased the mortality in patients with CDAD, including IBD patients, and concluded that the mortality of patients with CDAD on corticosteroids was significantly higher than the mortality of patients with CDAD not on corticosteroids, where both in corticosteroid users and non-users, patient age was an independent risk factor for mortality in the multivariate analysis [70]. Nguyen et al. investigated the discharge summaries of IBD patients in US National Inpatient Sample and reported that UC patients had higher prevalence and mortality of *C. difficile* infection than those with CD, non-IBD gastroenterology or general medical patients [71].

Gupta et al. reported that in their retrospective analysis of herpes zoster infections among IBD patients in the UK General Practice Research Database, both UC and CD patients had higher incidences than in the general population. In a nested case–control study including 451 IBD patients and 1787 controls [72], use of corticosteroids and immunosuppressive medications was presented to be associated with zoster which also showed, compared with people aged 5–44, a roughly two- to threefold increased risk among patients aged 45–64, and a fourfold increased risk among patients aged 65 and older. Furthermore, in Asia, Tsai et al. investigated the Research Database of Taiwan National Health Insurance and reported that male IBD patients had a higher risk for herpes zoster in age groups of 35–44 and 65 and more [73]. In a retrospective analysis of zoster among UC patients who had participated in the tofacitinib development programs, age over 65 years and refractoriness to anti-TNFα treatment were both reported to be independent risk factors [62].

On the other hand, the safety and efficacy of vedolizumab, anti-α_4_β_7_ integrin, in UC patients were similar for all age groups in a subgroup analysis of GEMINI-1, phase III trials [74].

## Surveillance in elderly UC patients

The risk for colorectal cancer is elevated in UC patients with long-term disease duration, but it is not clear whether the risk for colorectal cancer will increase in the elderly UC itself [49, 75]. There is no evidence that the risk for colorectal cancer is different between elderly and non-elderly UC patients [51]. Thus, there is no reason to change the surveillance method by age of disease onset.

However, in elderly UC, there are many cases whose onset time is unclear because of vague clinical symptoms [76], and it is possible that a long period of time has passed since disease onset at the time of diagnosis. Thus, it is necessary to start the surveillance program earlier in the patients whose onset time is unclear.

Colorectal cancer surveillance should be carried out in consideration of the balance between benefit and risk such as age, life prognosis, complication of other diseases including malignant tumors other than colorectal cancer, and risk for endoscopy [77].

## General attention of medical therapy for elderly UC patients

### Efficacy of medical therapy for elderly UC patients

Despite a few reports comparing the efficacy of individual medical treatments in non-elderly UC patients to that in elderly UC patients, there is no clear evidence that the efficacy obtained in non-elderly UC patients cannot be expected in elderly UC patients [7]. Therefore, all medical treatments are thought to be potential treatment options in elderly UC patients as well. In fact, medical treatments used in non-elderly UC patients are also selected for elderly UC patients in Japan [6, 22].

However, when considering medical treatments in elderly UC patients, one should pay attention to the interaction between comorbid diseases and their treatments; furthermore, a treatment needs to be decided whilst considering the characteristics of each patient, including susceptibility to infection and cancer risk. In fact, the frequency of adverse events such as infection and tumorigenesis is high in elderly UC patients who are treated with corticosteroids, thiopurine agents, or biological agents, and careful attention should be paid when administering these drugs [7, 76, 78]. Therefore, when selecting a medical treatment for elderly UC patients, risks associated with treatment should be considered on a case-by-case basis, especially as many new treatment options for IBD become available in the future [79]; dosages may have to be kept low in elderly UC patients and one should note that the efficacy may consequently decrease.

Moreover, in elderly patients with severe or intractable UC, one should perform medical treatments carefully considering various comorbidities and reduced physiological reserves, and always keep in mind not to miss an opportunity to refer for surgery in a proper timing.

The standard therapy for UC, 5-ASA agents, can be safely used in elderly UC patients; the efficacy in elderly patients was reported to be similar to that of non-elderly patients in most studies [78]. In studies of both elderly UC patients and elderly individuals without UC, the tolerance to topical formulations in enemas may decrease due to the decreased function of the anal sphincter [76].

Although there has not been a detailed study to examine the efficacy of corticosteroids in elderly UC patients, the efficacy is thought to be similar to that in non-elderly UC patients [80]. The rate of corticosteroid administration in elderly UC patients varies depending on studies [13, 18]. A nationwide survey in Japan reported a high rate of corticosteroid use in elderly UC patients [6].

The rate of thiopurine use in elderly UC patients is not high worldwide [18], including in Japan [22]. There have been no reports comparing the efficacy of thiopurine agents in elderly UC patients to that in non-elderly UC patients; however, the efficacy is thought to be similar [80]. Conversely, the risks of infection and malignant disease such as lymphoma have been observed to increase in elderly UC patients [81].

With respect to anti-TNF-α antibody agents, a study reported that in elderly UC patients treated with anti-TNF-α antibody agents, the rate of clinical improvement was lower than that in non-elderly UC patients during a short period (week 10 of administration); however, it was equal over a long period (over 6 months), suggesting that a certain period of time may be required until a curative effect is evident in elderly UC patients [82]. In contrast, it was reported that the therapeutic effect was low at 6 months after the initiation of administration and the rate of discontinuation was reported to be high in elderly UC patients [83], which may be particularly due to vulnerability to infectious complications requiring hospitalization.

The effect of therapeutic cytapheresis (CAP) in elderly UC patients is reported to be similar to that in non-elderly UC patients [84], whereas there is a contradictory report showing attenuation in its effect in elderly UC patients [85]. Thus, the efficacy of CAP in elderly UC is not consistent.

The efficacy of tacrolimus in elderly UC patients was only shown in a number of case reports and it has not been fully examined [86, 87]. Therefore, the difference in its efficacy between elderly and non-elderly patients remains unclear.

The efficacy of vedolizumab, an anti-α_4_β_7_ integrin antibody agent, in elderly UC patients was reported to be similar to that in other age groups in the sub-analysis, grouped by age (< 35 years, 35–54 years, and ≥ 55 years), of a phase III trial (GEMINI-1) [74].

There have been no reports on the efficacy of tofacitinib, a JAK inhibitor, in elderly UC patients. However, the sub-analysis of interview forms for pharmaceutical approval in the phase III trial of remission induction/maintenance therapies conducted in Japan and elsewhere has shown similar efficacy in elderly and non-elderly UC patients [88].

### Polypharmacy and drug interactions among elderly UC patients

Basically, there are no drugs that cannot be used for treatment of elderly UC [11, 89]. However, use of multiple drugs due to many concomitant diseases in addition to low organ reserve capacity will increase the risk for adverse drug reactions [11].

On the other hand, “polypharmacy” is of particular concern in the elderly due to the high possibility of difficulty in self-manage medication, leading to decreased adherence rates. Elderly patients with several comorbidities take multiple drugs (more than 5 drugs on average). Reportedly, 25% of the patients consume more than 6 types of drug [89]. Therefore, in elderly IBD patients with comorbidities, it is necessary to pay attention to the lower compliance rate and drug interaction caused by multiple drug therapy [89]. It has been shown that the clinical activity of IBD significantly increased in elderly depressive IBD patients through reduced medication adherence [90].

The risk for drug interaction rises according to the number of medicines prescribed: 13% for 2 or more kinds, 38% for 4 kinds, and 82% for 7 kinds [89]. Among them, the anticoagulant effect of warfarin potassium needs special attention. The mesalazine formulation enhances the anticoagulant effect because it inhibits the metabolism of warfarin potassium [91]. Corticosteroids attenuate the anticoagulant action of warfarin potassium by its coagulation accelerating action. Thiopurine formulation may also reduce the anticoagulant action of warfarin potassium [92]. In addition, allopurinol enhances thiopurine efficacy [93]. The mesalazine formulation also may enhance thiopurine efficacy by inhibiting thiopurine methyl transferase activity [94]. There is a possibility that the risk for bone marrow suppression and infectious diseases may be further enhanced especially in elderly UC patients.

Corticosteroid-treated elderly IBD patients have been reported to have a significantly increased risk for developing osteoporosis-related bone fractures, changes in mental state such as depression, deterioration of diabetes, hypertension, and glaucoma [95]. As the clinical course of IBD in these patients may be adversely affected, it is of utmost importance to limit the dose of corticosteroids in elderly IBD patients with the comorbidities.

In elderly individuals, although the rate of consuming anticoagulants and antiplatelet drugs for coronary artery disease, heart disease, and cerebrovascular disease is high, there is no evidence that antiplatelet therapy increases the frequency of IBD relapses. In a survey of 41 IBD patients who consumed aspirin and clopidogrel for coronary artery disease, there was no difference in the frequency of IBD relapses as compared with that of the control group. Indeed, patients treated with antiplatelet therapy showed an 11% reduction in IBD relapse [96]. Therefore, there is no reason to support the discontinuation of aspirin medication in IBD patients with cardiovascular complications [97, 98].

## Medical therapy for elder UC patients

### Steroids

Administration of adrenocorticosteroids (steroids) is recommended for the treatment of moderate-to-severe UC patients [9]. However, in using steroids for elderly UC patients, it is of particular importance to pay attention to infectious diseases in addition to side effects such as osteoporosis, osteonecrosis, edema, and cataracts. Generally, elderly people have a higher risk for infectious diseases due to an age-related decline in immune cell function and physical performance. Toruner et al. [55] reported that the use of corticosteroids is an independent risk factor for opportunistic infections in elderly IBD patients. Brassard et al. [69] reported that there was a correlation between the use of steroids and the development of a severe infectious disease in elderly-onset IBD patients. Komoto et al. reported that the proportion of patients using corticosteroids was significantly higher in EOUC patients than in NEOUC patients [6]. With regard to infectious diseases, Gupta et al. reported that the incidence rate of herpes zoster was higher among elderly IBD patients compared to healthy individuals. Furthermore, the risk was high among patients who used corticosteroids or immunomodulators [72]. From the cohort study conducted in France including 472 EOUC patients [15], risk factors for a colectomy in EOUC patients were investigated, which showed that the extent of disease distribution and the history of corticosteroid use were significant risk factors. However, by multivariate analysis, only the history of steroid use was found to be a significant risk factor. From these reports, we should pay attention not to administer corticosteroids to elderly UC patients for a prolonged period.

### Thiopurine

Thiopurine (azathioprine, 6-mercaptopurine) is effective and safe for maintaining remission of UC, and its effectiveness has been reported in Japan [99, 100]. Sustained thiopurine use of more than 1 year in EOUC patients was associated with a 70% reduction in risk for colectomy [hazard ratio (HR) 0.30; 95% CI 0.15–0.58] [101]. A combination of thiopurine and infliximab showed a twofold greater effect for steroid-free remission than thiopurine monotherapy in the UC SUCCESS trial (39.7% vs. 22.1%, *p* = 0.017) [102]. However, the benefit of combination therapy for the elderly has not been confirmed as elderly patients were not included in this trial. Combination immunosuppression therapy was a significant predictor of anti-TNF cessation with a 2.2-fold increase in the likelihood in elderly anti-TNF users [83]. Thus, it is necessary to remain cautious about the use of combination therapy in the elderly.

Regarding adverse effects related to thiopurine, there was higher incidence of hepatotoxicity and a lower incidence of acute pancreatitis in the elderly-onset IBD patients [103]. In addition, older age alone is considered as a risk for opportunistic infection [55]. Furthermore, patients receiving thiopurine also have a greater risk for opportunistic infections (i.e., herpes simplex virus, HZV, CMV, and Epstein–Barr virus) and tuberculosis (TB) [57, 104].

The use of thiopurines has been shown to be associated with an increased risk for lymphoproliferative disorder in several studies [9, 10, 105–107]. The CESAME study, a multicenter prospective cohort study from France, showed that continuing thiopurine therapy, older age, and a longer duration of IBD were the main risk factors for lymphoproliferative disorder [9, 10, 106]. Meta-analysis showed that the risk for lymphoma rises approximately 4–5 times in IBD patients treated with thiopurines [10, 105, 107]. The risk for lymphoma increased especially in IBD patients older than 50 years [108]. The use of anti-TNF monotherapy (HR 2.60; 95% CI 1.96–3.44) and thiopurine monotherapy (HR 2.41; 95% CI 1.60–3.64) has a significantly increased risk for lymphoma, respectively, and this risk becomes approximately 2 times higher with combination therapy than each of these treatments used alone according to the French National Health Insurance claim database [109]. However, a multicenter retrospective study in Japanese patients with IBD suggested that thiopurine may not be a risk factor of hematologic malignancies; therefore, further examination is necessary for Japanese IBD patients [110].

An increased risk for non-melanoma skin cancer associated with thiopurine use has been found in the American database (the LifeLink Health Plan Claims Database) with approximately 110,000 IBD patients from 1997 to 2009 [111]. In addition, present thiopurine exposure increases the risk for non-melanoma skin cancer, particularly in the elderly Caucasian population [112]. However, there is a lack of data in the Japanese IBD patients regarding association between thiopurine use and non-melanoma skin cancer.

### Tacrolimus

Tacrolimus is an immunosuppressant known to inhibit the production of cytokines including IL-2 [113] used for solid organ transplantation [114], rheumatoid arthritis [115], and refractory UC [116].

While the effectiveness of tacrolimus in the induction of remission of UC has been demonstrated [117, 118], side effects, such as nephrotoxicity, neurotoxicity, hypertension, and secondary infections, are known to develop depending on trough concentration. [118]. The effectiveness and safety of tacrolimus in elderly UC patients has only been detailed in case reports [86, 87, 119].

Elderly UC patients are susceptible to various complications, such as renal insufficiency and hypertension, and are also prone to infections [7, 11, 59]. Furthermore, infection is a risk factor of hospitalization [68]. In this regard, the rate of opportunistic infections increases in patients with IBD after the age of 50 [55, 57], which is considered a critical factor leading to death [11]. Cyclosporin, another calcineurin inhibitor, is not recommended for elderly UC patients older than 60 years as it may aggravate complications [120, 121]. The rate of cyclosporine and tacrolimus use in European patients ≥ 65 years of age is only < 0.5% (2004–2009) [122]. When using calcineurin inhibitors in elderly UC patients, it is necessary to accurately evaluate renal and other organ functions [86, 123]. In addition, in patients treated with several immunotherapeutic agents, including tacrolimus, administration of prophylactic agents against opportunistic infections is highly recommended [59, 119]. Tacrolimus is metabolized by cytochrome P450 3A enzymes. Older patients often use several drugs for various complications, and it is important to check for potential interactions with agents that have the same metabolic pathway, such as nifedipine (Ca antagonist) and lansoprazole (PPI).

There is a paucity of information on optimal tacrolimus dosage and target trough concentration in the elderly, even in diseases out with UC. Furthermore, the initial dosage of tacrolimus has not yet been set for elderly kidney transplant recipients relative to their younger counterparts [124]. Thus, it is important to monitor tacrolimus blood trough concentrations in the elderly during the maintenance phase of treatment [124]. It has been reported that the infection rate was within an acceptable range in patients, with an average age of 61 years ranging from 50 to 77 years old, who were treated by tacrolimus for renal transplantation [125]. In patients with rheumatoid arthritis, the routinely used dose of tacrolimus is 3 mg/day, whereas the recommended starting dose for those aged more than 65 years is 1.5 mg/day [115]. Aging beyond 65 is associated with increased risk for renal dysfunction, thus treating elderly patients with tacrolimus is a risk factor of nephropathy [126].

### Anti-TNFα agents

Currently, 3 types of anti-TNF agents (i.e., infliximab, adalimumab, and golimumab) can be used to treat patients with moderate-to-severe UC in whom existing drug therapy has been ineffective.

In a multicenter, prospective study conducted in Italy for 10 years, the anti-TNF agents infliximab and adalimumab were administered to 2,475 and 604 IBD patients, respectively, to compare the occurrence of infections, malignant neoplasms, and death. The comparison of elderly and non-elderly findings revealed the rates of infection (13% vs. 2.6%), malignant neoplasms (3% vs. 0%), and death (10% vs. 1%), suggesting that occurrence rates were elevated in elderly patients [19]. In addition, a study reported that the risk for malignant neoplasms and infections among patients aged > 65 years who received anti-TNF agents was 4.7 times higher than those among non-elderly patients [82]. Hence, anti-TNF agents should be administered to elderly patients with caution.

The risk for TB increases with age and anti-TNF administration. Therefore, TB should be screened for before treating with immunomodulators or biologics [127]. Furthermore, treating adult patients with CD with anti-TNF agents alone or combined with immunomodulators increases the risk for developing non-Hodgkin’s lymphoma [109]. Moreover, the risk elevation tends to occur more often among male and elderly patients [128, 129].

Concerning the incidence of death related to anti-TNF agents, a study comparing 734 anti-TNF-treated and 666 anti-TNF-untreated patients reported that the age at anti-TNF induction was the single independent factor causing death [130]. Furthermore, 75% of infliximab-related deaths occurred among patients aged > 65 years. It is reported for Crohn’s disease that patients who died had the disease for a long period of time (15–26 years), had severe disease activity and complications, and used immunomodulators in combination [131]. On the other hand, the risk for severe infection does not change between treatment with anti-TNF agents alone and with a combination of anti-TNF agents and immunomodulators [19].

Deaths due to cardiovascular events that developed after the anti-TNF administration have been reported [82]. Therefore, the cardiac functions should be screened before administering anti-TNF agents. Anti-TNF could be used for patients with an ejection fraction > 50% and well-compensated heart failure (NYHA class I–II). The use of anti-TNF drugs for patients having severe heart failure (NYHA class III–IV) elevates mortality rate, rendering their use unsuitable [132]. Anti-TNF agents have been shown to increase death related to heart failure among patients with rheumatoid arthritis [132]. Hence, cardiac function should be monitored in patients with heart failure.

### Cytapheresis

There are a few reports on the efficacy and safety of CAP in elderly UC patients [84, 85, 133]. These reports highlighted the safety of CAP in elderly patients because the incidence of adverse events (AEs) for CAP is not different between elderly patients and the non-elderly patients with UC. Komoto et al. determined in their retrospective multicenter cohort study (*n* = 847) that the incidence of AEs was 8.0% in the elderly UC patients and 10.5% in non-elderly patients [133]. They also reported that most AEs were mild events such as nausea or a mild decrease in platelet counts; severe AEs such as severe infection or thrombosis were not observed. Moreover, Ito et al. reported that CAP is a safe therapy for elderly UC patients as there were only mild AEs such as headache or allergic reaction to anticoagulants [84].

## Surgical treatment in elderly UC patients

### Indication of surgical treatment

In general, the absolute indications for surgery in patients with UC include bowel perforation, uncontrollable bleeding, toxic megacolon and colitis-associated colorectal cancer. The relative indications for surgery include refractory disease with failure of medical management.

Elderly UC patients are more frequently operated on due to severe or fulminant disease compared to non-elderly patients [134, 135]. Because elderly UC patients have few subjective symptoms even in severe or fulminant disease, it is difficult to judge the indication of surgery in elderly patients. Therefore, the disease activity and patient condition should be more carefully evaluated in elderly UC patients.

In elderly patients, surgery with the permanent colostomy is more often selected, because the sphincter-preserving procedure cannot be performed due to deterioration of anal sphincter function [134, 135].

### Timing of surgical treatment

Elderly people often have concomitant complications, such as cardiovascular disease and diabetes, which could affect surgical prognosis [134]. Therefore, surgery should be performed before the general status gets worse [136].

Elderly UC patients with pneumonia and thrombosis during hospitalization increases postoperative mortality, especially in the case of emergent surgery [134].

The timing of elective surgery does not have to be changed between non-elderly and elderly UC patients with relatively good general condition in the case of the patients with early colitis-associated colorectal cancer.

On the other hand, early determinations for surgery should be considered in patients with severe complications, with severe or fulminant disease activity, or refractory to medical treatments, because their postoperative course was worse compared to non-elderly patients if the timing of surgery is the same [7, 136]. In elderly patients, the ratio of patients whose ADL has weakened after the surgical procedure was high, leading to development of pneumonia, difficulty in ambulation, and the deterioration of swallowing. Thus, elderly patients need to be determined for the early surgical treatment before their ADL get lower.

### Selection of surgical procedure

Elderly UC patients tend to decrease general condition, organ function, anal sphincter function, or ADL compared with non-elderly UC patients. Under the consideration of these factors, surgical procedures should be determined to be safe and maintain patients’ quality of life [7]. Preoperative sufficient informed consent is also important.

Following 5 surgical procedures are generally selected: (1) ileal pouch anal anastomosis with mucosectomy (hand-sewn IPAA), (2) stapled ileal pouch anal anastomosis (stapled IPAA), (3) ileorectal anastomosis (IRA), (4) total proctocolectomy with end ileostomy (TPC), and (5) subtotal colectomy with end ileostomy, mucous fistula of the sigmoid colon, or Hartmann’s operation.

Some papers reported that sphincter-preserving surgery (hand-sewn or stapled IPAA) resulted in good functional outcome in elderly UC patients, if the patients had a good anal sphincter function and good ADL preoperatively [134, 137–140]. On the other hand, it is also reported that sphincter-preserving surgery for elderly UC patients tended to increase long-term postoperative complications [139] and decrease bowel function [137, 140, 141]. Functional outcome after stapled IPAA is reported to be better compared with that after hand-sewn IPAA because anal canal tissue is preserved in stapled IPAA [137, 142].

In elderly UC patients with impaired sphincter function or lower ADL, total colectomy + IRA or TPC is usually selected in consideration of postoperative functional outcomes. Stapled IPAA and IRA preserve the diseased mucosa; therefore, it is important to pay attention to the risk for relapse or cancer [9]. In the case of emergent surgery with poor general conditions, less invasive surgical procedures such as subtotal colectomy with end ileostomy, mucous fistula of the sigmoid colon, or Hartmann’s operation should be selected first [16]. Surgical procedures accompanied with stoma construction are more often selected in elderly UC patients compared with non-elderly UC patients [16, 143].

In cancer cases, surgical procedures for elderly UC patients should be carefully determined in consideration of performance status, extension of colitis, organ function, ADL, advance of cancer, and colitis-associated cancer or sporadic cancer. In the case of colitis-associated cancer, total proctocolectomy is usually selected in consideration of the risk for cancer at the other colorectal site. If the patients had a good anal sphincter function and good ADL, hand-sewn IPAA can be offered. On the other hand, TPC with permanent end ileostomy is usually selected if the patients impaired sphincter function, lower ADL, or advanced cancer located in lower rectum or anal canal. Stapled IPAA is not usually selected in consideration of the risk for cancer at the remnant anal canal; however, it is sometimes selected in comprehensive consideration of patients’ condition. When stapled IPAA is performed, close follow-up for the remnant anal canal is required [141]. If the general condition is too poor to undergo total colectomy, ordinary segmental colectomy is performed in consideration of safety and postoperative QOL. In the case of sporadic cancer, ordinary segmental colectomy can be also selected. In these cases, postoperative follow-up for the remnant colorectal tissue is required.

### Postoperative complications and prognosis after surgical treatment

It is generally recognized that older age regardless of disease is an independent risk factor for surgical mortality and morbidity [144]. However, some cohort studies of patients with UC have suggested that those were similar among several age groups [143]. Nevertheless, it is commonly agreed that infectious complications and venous thrombosis, known to be increasing in older patients regardless of surgical treatments, must be considered during treatment for UC [19, 83, 130]. Although pouch surgery for UC has been shown to be safe, and surgical morbidity and mortality are similar regardless of age, emergent surgery and concomitant complications with other systematic diseases, such as heart disease or pulmonary disease, are shown to be prognostic factors in the surgical treatments for older patients [20, 137, 138, 145, 146]. In a study conducted in Japan, surgical mortality in the older group who received an emergency procedure was at a rate of 26.7% within 30 postoperative days [16], though surgical morbidity and mortality were similar between patients of older age and younger age.

In conclusion, the rates of surgical morbidity and mortality may be increasing in older patients with UC in association with the method of surgical procedure, or concomitant complications with other systematic diseases. Notably, it is important to highlight that emergency surgery for older patients elevates the risk for surgical morbidity and mortality.

### Influence of surgical treatment on anal function and daily life

Generally, anal function deteriorates with aging, while it has also been reported that it can progressively worsen and fecal incontinence increases after ileal pouch anal anastomosis for UC in older patients [147]. Another study noted that the incidence of night time fecal incontinence may be higher in patients older than 65 years as compared to younger patients, though stool frequency was not found to change with aging [138]. On the other hand, no relationship between aging and fecal incontinence after pouch surgery was seen in several other reports [139, 148, 149]. Although opinions vary, an interesting study recommended a stapled, not hand-sewn, anal anastomosis to maintain anal function in older patients [89].

In a study conducted in Japan, the prevalence of stapled anal anastomosis was higher in patients older than 60 years as compared to the younger group [16]. They noted that a stapled anal anastomosis or permanent ileostomy had tendencies to be used as a standard surgical procedure for elderly patients. As for anal anastomosis, another report noted that anal function after a stapled anastomosis was similar between non-elderly and elderly patients [134]. However, it is important to consider operative method of anastomosis for older patients, because anal function deteriorates following a hand-sewn procedure in association with aging, even though good QOL can be maintained in most cases [140].

A permanent ileostomy can contribute to maintain the good QOL, such as with a restorative proctocolectomy. In some older patients, there are no restrictions or issues with defecation following a permanent ileostomy, with improved QOL similar to that seen in younger patients or after a restorative proctocolectomy [150]. In cases with mild inflammation or with low risk for developing colitis-associated cancer, an IRA is occasionally recommended for older patients to preserve anal function [89].

In conclusion, even though pouch function may become worse in older patients with UC, QOL can be maintained at the same level as prior to surgery with a proper surgical procedure.

## Abbreviations

AEs: Adverse events
CAP: Cytapheresis
CD: Crohn’s disease
*C. difficile*: *Clostridioides* (*Clostridium*) *difficile*
CDAD: *Clostridium* (*Clostridioides*) *difficile*-associated diarrhea
CMV: Cytomegalovirus
ECCO: European Crohn’s and Colitis organization
HBV: Hepatitis B virus
HZV: Herpes zoster virus
IBD: Inflammatory bowel disease
IPAA: Ileal pouch anal anastomosis
IRA: Ileorectal anastomosis
NSAIDs: Non-steroidal anti-inflammatory drugs
PPIs: Proton pump inhibitors
SCAD: Segmental colitis associated with diverticulosis
SRUS: Solitary rectal ulcer syndrome
TB: Tuberculosis
TNF: Tumor necrosis factor
TPC: Total proctocolectomy with end ileostomy
UC: Ulcerative colitis
5-ASA: 5-Aminosalicylic acid
LEUC: Longstanding elderly UC (elderly UC patients who had first diagnosed as having UC at a young age)
EOUC: Elderly-onset UC (elderly UC patients with onset of UC at a late age)
Elderly UC: UC patients including LEUC and EOUC
NEOUC: Non-elderly-onset UC
