# Correction to: Management of elderly ulcerative colitis in Japan

**DOI:** 10.1007/s00535-019-01606-5

**Published:** 2019-08-07

**Authors:** Masaaki Higashiyama, Akira Sugita, Kazutaka Koganei, Kenji Wanatabe, Yoko Yokoyama, Motoi Uchino, Masakazu Nagahori, Makoto Naganuma, Shigeki Bamba, Shingo Kato, Ken Takeuchi, Teppei Omori, Tomohisa Takagi, Satohiro Matsumoto, Mitsuo Nagasaka, Shintaro Sagami, Kazuya Kitamura, Takehiko Katsurada, Ken Sugimoto, Noritaka Takatsu, Masayuki Saruta, Toshiyuki Sakurai, Kazuhiro Watanabe, Shiro Nakamura, Yasuo Suzuki, Ryota Hokari

**Affiliations:** 1 Department of Internal Medicine, National Defense Medical College, 3-2 Namiki, Tokorozawa, Saitama 359-8513 Japan; 2 Inflammatory Bowel Disease Center, Yokohama Municipal Citizen’s Hospital, Yokohama, Kanagawa Japan; 3 Department of Intestinal Inflammation Research, Hyogo College of Medicine, Nishinomiya, Hyogo Japan; 4 Department of Inflammatory Bowel Disease, Division of Surgery, Hyogo College of Medicine, Nishinomiya, Hyogo Japan; 5 Department of Gastroenterology and Hepatology, Tokyo Medical and Dental University, Tokyo, Japan; 6 Division of Gastroenterology and Hepatology, Department of Internal Medicine, Keio University School of Medicine, Tokyo, Japan; 7 Division of Clinical Nutrition, Shiga University of Medical Science, Otsu, Shiga Japan; 8 Department of Gastroenterology and Hepatology, Saitama Medical Center, Saitama Medical University, Saitama, Japan; 9 Division of Gastroenterology and Hepatology, Department of Internal Medicine, Toho University Sakura Medical Centre, Sakura, Chiba Japan; 10 Institute of Gastroenterology, Tokyo Women’s Medical University, Tokyo, Japan; 11 Molecular Gastroenterology and Hepatology, Graduate School of Medical Science, Kyoto Prefectural University of Medicine, Kyoto, Japan; 12 Department of Gastroenterology, Saitama Medical Center, Jichi Medical University, Saitama, Japan; 13 Department of Gastroenterology, Fujita Health University School of Medicine, Toyoake, Aichi Japan; 14 Center for Advanced IBD Research and Treatment, Kitasato University Kitasato Institute Hospital, Tokyo, Japan; 15 Department of Gastroenterology, Kanazawa University Hospital, Kanazawa, Ishikawa Japan; 16 Department of Gastroenterology and Hepatology, Hokkaido University Graduate School of Medicine, Sapporo, Hokkaido Japan; 17 First Department of Medicine, Hamamatsu University School of Medicine, Hamamatsu, Shizuoka Japan; 18 Department of Gastroenterology, Fukuoka University Chikushi Hospital, Chikushino, Fukuoka Japan; 19 Division of Gastroenterology and Hepatology, Department of Internal Medicine, The Jikei University School of Medicine, Tokyo, Japan; 20 Department of Surgery, Tohoku University Graduate School of Medicine, Sendai, Miyagi Japan; 21 Inflammatory Bowel Disease Center, Toho University Sakura Medical Centre, Sakura, Chiba Japan

## Correction to: J Gastroenterol (2019) 54:571–586 10.1007/s00535-019-01580-y

The article “Management of elderly ulcerative colitis in Japan”, written by Masaaki Higashiyama, Akira Sugita, Kazutaka Koganei, Kenji Wanatabe, Yoko Yokoyama, Motoi Uchino, Masakazu Nagahori, Makoto Naganuma, Shigeki Bamba, Shingo Kato, Ken Takeuchi, Teppei Omori, Tomohisa Takagi, Satohiro Matsumoto, Mitsuo Nagasaka, Shintaro Sagami, Kazuya Kitamura, Takehiko Katsurada, Ken Sugimoto, Noritaka Takatsu, Masayuki Saruta, Toshiyuki Sakurai, Kazuhiro Watanabe, Shiro Nakamura, Yasuo Suzuki, and Ryota Hokari was originally published Online First without open access. After publication in volume 54, issue 7, page 571–586 the Japanese Society of Gastroenterology requested that the article be Open Choice to make the article an open access publication. Post-publication open access was funded by the Japanese Society of Gastroenterology. The article is forthwith distributed under the terms of the Creative Commons Attribution 4.0 International License (http://creativecommons.org/licenses/by/4.0/), which permits use, duplication, adaptation, distribution and reproduction in any medium or format, as long as you give appropriate credit to the original author(s) and the source, provide a link to the Creative Commons license and indicate if changes were made.

The original article has been updated.

